# Predicting Intracerebral Hemorrhage Patients' Length-of-Stay Probability Distribution Based on Demographic, Clinical, Admission Diagnosis, and Surgery Information

**DOI:** 10.1155/2019/4571636

**Published:** 2019-01-27

**Authors:** Li Luo, Xueru Xu, Yan Jiang, Wei Zhu

**Affiliations:** ^1^Business School, Sichuan University, Chengdu 610065, China; ^2^West China Hospital, Sichuan University, Chengdu 610041, China

## Abstract

The vast majority of patients with intracerebral hemorrhage (ICH) suffer from long and uncertain length of stay (LOS). The aim of our study was to provide decision support for discharge and admission plans by predicting ICH patients' LOS probability distribution. The demographics, clinical predictors, admission diagnosis, and surgery information from 3,600 ICH patients were used in this study. We used univariable Cox analysis, multivariable Cox analysis, Cox-variable of importance (Cox-VIMP) analysis, and an intersection analysis to select predictors and used random survival forests (RSF)—a method in survival analysis—to predict LOS probability distribution. The Cox-VIMP method constructed by us effectively selected significant correlation predictors. The Cox-VIMP RSF model can improve prediction performance and is significantly different from the other models. The Cox-VIMP can contribute to the screening of predictors, and the RSF model can be established through those predictors to predict the probability distribution of LOS in each patient.

## 1. Introduction

Intracerebral hemorrhage (ICH) is one of the most detrimental subtypes of stroke and accounts for 10–15% of all strokes [1]. According to the statistics, the mortality of ICH is 30–50% every year [2]. The incidence of ICH was 10–30 cases per 100,000 people/year in 2001 and is expected to double by 2050 [3]. After the onset of ICH, patients need hospitalization and some of them need surgical treatment. This disease places a heavy burden on the family and society. The lifetime cost of ICH is more than $123,500 [4], and the mean cost per inpatient day is $1,396 [5]. As a measure of resource use, LOS is strongly associated with patient cost, explaining 72–82% of the variation in cost [5]. Intracerebral hemorrhage hospitalization is characterized by two factors: long LOS and uncertainty in LOS. With regard to long LOS, the average LOS is longer for patients with ICH than for patients with other diseases [6]. As a result, the bed turnover rate is low, which leads to longer hospital stays and longer admission queues. With regard to uncertainty in LOS, patients with similar disease conditions at the time of admission will have differences in LOS, which increases the chances of failing to predict the LOS and failing to serve the next patient in a timely manner. Random uncertainty in bed usage can lead to penalty costs for resource scheduling failures (e.g., patient waiting cost or idle bed cost).

Bed management (BM) serves as a very important resource management tool [7, 8] for facing these challenges. The aims of BM are to reduce the vacancy rates of beds, to improve utilization rates, to serve more patients, and to enhance the social medical supply. In clinical practice, the problems generated by long and uncertain LOS are the main targets for improvement in BM. Hence, prediction of LOS is a primary reason for implementing BM [9]. Predicting LOS can improve the utilization rate. Healthcare managers can avoid unnecessary discharge waiting time and make more beds available through rational arrangement of the patient's discharge, allowing the bed to serve more patients and improving bed utilization rates. Moreover, predicting LOS can reduce the vacancy rate of beds. Healthcare managers make admission plans in advance, and the number of admitted patients per day is based on the number of patients discharged that day [10]. Admission service center staff can give patients who are waiting for admission a phone call about their admission time in advance. The waiting patients can prepare ahead of time and arrive at the hospital immediately [11]. Otherwise, beds will be empty until the patients arrive in the hospital.

However, in practice, the data-handling capacity of the human brain is lower than that of data analyzed by computers, especially high-dimensional data. It is difficult to accurately estimate in advance the number of patients who will be discharged every day using only the experiences of healthcare managers. The prediction results are easily affected by external factors. Hence, when faced with high-dimensional data, data analysis is regarded as an effective way to predict LOS. Past studies [12–17] have constructed prediction models to estimate general LOS, but few have been conducted on ICH patients' LOS. Russell et al. [4] used the 2002 Healthcare Cost and Utilization Project Nationwide Inpatient Sample to assess hospital LOS among ICH patients. The study used bivariate analyses to select patients' variables associating with LOS. The predictors extracted in the study by Russell et al. were referenced in the present study. However, the authors only selected variables and did not predict LOS using the model. A review of the available literature showed that even though few studies focused on LOS among ICH patients', there were some LOS prediction studies for other diseases [12, 15–17] or specific departments [18], such as the intensive care unit [13] and emergency department [14]. LOS prediction can be divided into three categories: specified value prediction, classification prediction, and probability distribution prediction.

First, some studies have directly predicted LOS. Nagarsheth et al. [15] used the data of pediatric patients who were injured in all-terrain vehicle accidents from January 2000 to December 2009 to create a simple mathematical model to calculate LOS. Caetano et al. [16] focused on the case study of a Portuguese hospital and used a data-driven predictive model to obtain LOS. Rowan et al. [17] used an artificial neural networks model to predict LOS after cardiac surgery. The specified LOS is of great significance in the process of clinical treatment. However, because of the uncertainty in patients' LOS, it is unreasonable to predict whether an event will happen at a certain time in the future. Patients with the same disease conditions at the time of admission will have differences in LOS. Hence, it is controversial to predict an actual number for LOS.

Second, some studies treated LOS prediction as a classification prediction [18–20]. Tanuja et al. [18] compared four different data mining techniques (multilayered backpropagation neural network, naive Bayes classifier, K-nearest-neighbors method, and J48 class of C4.5 decision tree) in predicting LOS using three time intervals (0–7 days, 7–14 days, and 15–30 days). Hachesu et al. [19] divided LOS into three groups, according to different thresholds: LOS ≤ 5; LOS between 6 and 9; LOS > 10. Three classification algorithms (decision tree, support vector machines, and artificial neural network) and 36 input variables were used in the study by Hachesu et al. Because the distribution of hospital LOS is highly skewed and can vary in shape [20], a classification prediction model dividing LOS into different periods of time can reduce the impact of shapes on prediction accuracy. Usually, the greater the interval, the higher the accuracy of prediction. However, if the classification interval is too large, the results do not have adequate significance for BM. For example, if the LOS is predicted to be 5–10 days, it is difficult to determine the approximate discharge date. The classification interval is influenced by many factors, including researcher subjectivity. There is no definite criterion to divide the intervals, which leads to greater fluctuation in the accuracy of the final prediction. Thus, the method may sometimes not reach the expected accuracy.

Third, some studies have put forward predictions about LOS probability distribution [21, 22]. Predicting LOS probability distribution better captures the character of LOS [22]. With a probability curve, we can intuitively understand the trend of hospitalization probability of patients, which can facilitate bed management. Rauner et al. [22] analyzed the effects of the new Austrian performance-oriented inpatient payment system on discharge strategies of hospitals by investigating LOS distributions. Survival analysis is widely used in LOS distribution studies [23–25]. Thus, random survival forests (RSF), a commonly used method of calculation in survival analysis, has proven to be effective in predicting the probability distribution of LOS. Random survival forest is an ensemble tree method used in the analysis of right-censored survival data.

Most of the literature that predicts LOS uses demographic and clinical information [2, 6–8]. However, it is not sufficient to use only these two sources of information in developing a prediction model because this information does not reflect the patient's disease conditions. Therefore, we cannot ignore diagnostic and surgical information in the prediction of LOS and need to consider how to use this information effectively. As variables, hemorrhage locations and whether or not surgery was performed are indispensable predictors [6]. However, only using these data does not make full use of available information. In addition to hemorrhage locations, preexisting diseases can also affect LOS. The LOS is affected not only by whether surgery was conducted but also by preexisting diseases. Hence, attention should be paid to the preexisting diseases of each patient and to the details of the surgery process (if applicable). The more complicated the surgery details, the more serious the disease.

However, when the diagnostic and surgical information are included in the model, the data dimension is greatly increased. For a large number of predictors, to avoid overfitting, investigators should select the predictors that are significant correlation predictors. The process of selection of predictors is an indispensable part of the research. In research using the survival analysis model, many studies commonly used the Cox regression model to select variables [26, 27]. Li and Gui [26] developed a partial Cox regression method to screen microarray gene expression data for predicting the survival rate of future patients. Sierra Zúñiga et al. [27] used the Cox regression survival model to explore the association between covariates and LOS. Moreover, other scholars selected variables by calculating the value of importance (VIMP). Barnes et al. [28] used VIMP to find the most significant predictors in LOS prediction. Torisson et al. [29] used the survival analysis model to calculate the relative VIMP of factors in a mortality prediction model. However, the Cox model or VIMP calculation cannot eliminate the interaction between variables by itself. In fact, the combination of methods can avoid the influence of the relationship among variables on the prediction model and should be considered in research.

In this study, we aimed to select the significant predictors for ICH patients' LOS and estimate the probability distribution of LOS probability. Compared with other studies on ICH patients' LOS prediction, our study differs in that we focused on the LOS probability distribution, made full use of the patient's diagnostic and surgery information, and used the combined Cox methods to deal with high-dimensional data. The results of our research allow doctors to better understand which predictors affect LOS and comprehend the LOS probability distribution of each patient, and will thus aid healthcare managers in arranging the patient's discharge and admission plans. The results of our study may have practical significance for BM in the ICH department.

## 2. Methods

### 2.1. Data

The data for this study are sourced from the neurology department at West China Hospital, one of the best hospitals in China. Three thousand six hundred ICH patients were admitted to the hospital over a 36-month period, from January 1, 2014, to September 31, 2016. Predictors were divided into four categories: demographic predictors, clinical predictors, admission diagnosis, and surgery information. Demographic and clinical predictors, the common predictors in other research, include age, gender, marital status, occupation, ethnicity, payment type (general medical insurance, nonmedical insurance, and special medical insurance), doctor (attending doctors in the neurology department), admission type (emergency, outpatient, and others), and transfer information (whether or not the patient was transferred from another medical institutions). Admission diagnosis can be divided into three parts: the main diagnosis (the information description of the hemorrhage location), preexisting diseases, and the number of diseases. Surgery information can be divided into two parts: surgery details and the number of surgeries. It is worth noting that the surgery information is not of the surgeries undertaken during hospitalization, but the surgery details of the first operation after admission. According to the doctors' advice, a LOS over 100 days was abnormal data and could be disregarded. Furthermore, for some patients, the reasons for rehospitalization were different from the first time, and the number of such patients was very small. Thus, the sample for this study consisted of ICH patients who were hospitalized for the first time. After data preprocessing, 2,583 patients remained in the sample. These data are summarized in Table 1.

The average LOS for ICH patients was 12.6 days, and the average age of patients was 53.79 years. The sample consisted of 53.5% men and 46.5% women. Demographic information also produced statistics on marital status, occupation, and ethnicity. Payment type was divided into three parts: general medical insurance (26.8%), non-medical insurance (61.9%), and special medical insurance (11.3%). Patients with special medical insurance can receive services more quickly. These data involve 132 doctors working in the neurology department, who treat different types of patients. There are three admission types: emergency (89.6%), outpatient (7.5%), and other (0.8%). About 24.7% of patients had been transferred from other medical institutions, 71% had not, and this information was not recorded for 4.3% of the patients.

The individual differences in diagnostic and surgical information are vast. To resolve this issue, we used one-hot processing for collecting all the patients' diagnostic and surgical information and transformed this information into binary variables (0/1). The value of 0 indicated that the patient did not have the disease or the surgery procedure, and the value of 1 implies the opposite. For example, if a patient has hypertension, hypertension will be treated as a variable with the value 1. After one-hot processing of diagnosis data and surgical information, there were about 8,459 variables that could be used in the prediction model (550 diseases and 7,933 surgical details). Each patient had an average of 3 diagnoses, and there was an average of 3.6 surgical contents in the first surgery. The number of predictors was greater than the number of samples, which could have led to overfitting. Thus, the indispensable work of this study was to reduce data dimensions and select the most significant predictors of LOS.

### 2.2. Selection of Predictors

With dimensional data, the predictor-selection process is complex, and the number of predictors directly affects the accuracy of the prediction model. The Cox regression model, one of the most common methods for selecting predictors, was used in this study. To eliminate the interaction between predictors, three Cox combination schemes based on univariable Cox were proposed (i.e., multivariable Cox, Cox-VIMP, and intersection). In other words, univariable Cox was set as the baseline model and was compared with the other three Cox combination schemes. Based on the results of the four schemes, four prediction models were established. Finally, the performance of the prediction model was used to determine which scheme was most effective.

### 2.3. Scheme 1: Univariable Cox

The single-variable Cox regression model was used to test the independent contribution of each predictor.

(1) $$\begin{array}{c} h\left(t,X\right)=h_{0}\left(t\right)\exp\left(\beta_{i}X_{i}\right),\;i=1,2,3,\ldots ,m. \end{array}$$

The *h*(*t*, *X*) is the risk rate function, meaning the instantaneous death rate (incidence of events) of the variable *X* at the time of *t*. *β*_*i*_ is the partial regression coefficient of the independent variable and the benchmark risk rate of *h*(*t*, *X*) when the *X* vector is 0, which is an estimated parameter from the sample data. If a predictor's *P* value < 0.05, we treated this predictor as an effective one.

### 2.4. Scheme 2: Multivariable Cox

The predictors selected from the univariable Cox analysis did not remove the mutual influence between predictors. To remove the influence, a multivariable Cox regression model was used to remove some of the unnecessary predictors (*P* value > 0.05). This is a commonly used method to eliminate interaction effects. The predictors from univariable Cox analysis are taken into the multivariable Cox analysis.

(2) $$\begin{array}{c} h\left(t,X\right)=h_{0}\left(t\right)\exp\left(\beta_{1}X_{1}+\beta_{2}X_{2}+\cdots +\beta_{m}X_{m}\right). \end{array}$$

Predictors with *P* values < 0.05 were chosen as significant correlation predictors.

### 2.5. Scheme 3: Cox-VIMP

Based on the results of the univariable Cox analysis, the VIMP of each predictor could be calculated. The VIMP measures the contribution of each predictor in the prediction model—the greater the VIMP, the stronger the predictive power. In this study, we treated predictors with VIMP > 0, as an alternative important predictor.

The larger the predictor's VIMP, the greater the predictor's contribution. However, there is no definition of a value of VIMP at which and above which optimal results are guaranteed. In order to find the most effective predictors, we proposed a nested analysis. We sequenced predictors with VIMP > 0 by their VIMP and considered the nested sequence of prediction models, which starts with the top variable, followed by the model with the top two variables, then the model with the top three variables, and so on [30]. The *k*-fold cross-validation method was used to test the stability of these models. The optimal model was chosen using the pessimistic principle, which means the model with the maximum *C*-index (see description of Experimental Setup) minus standard deviation. Furthermore, the predictors in this model were treated as the significant predictors.

### 2.6. Scheme 4: Intersection

Predictors that were in all of the above three schemes were considered the significant predictors.

### 2.7. Experimental Setup

Taylor [30] verified that RSF can handle high-dimensional data well. It is reasonable for us to use RSF to deal with high-dimensional data. The calculation principle of RSF uses the self-help method (bootstrap) to extract *n*-tree bootstrap samples from the original data and spanning tree for each bootstrap sample, until the number of leaf nodes is not less than node size. In the present study, four models were constructed based on four sets of predictors: univariable Cox RSF model, multivariable Cox RSF model, Cox-VIMP RSF model, and intersection RSF model. Each model was run 100 times with 80% randomly taken samples (the model has a different training set in each time) as the training set, and the predictive performance value was calculated each time. If the differences in performance values between these 100 predictions were relatively small, the model be treated as repeatable and reasonable. After 100 runs, the average performance value was the final performance value of that model which was used to determine the optimal model. The paired samples *t*-test was used to test whether there were differences among the four models in the measured value. These analyses were conducted using R software (R Foundation for Statistical Computing, Vienna, Austria).

In order to evaluate models' performance, the measure of the RSF model was calculated by using the Harrel consistency index (*C*-index). The *C*-index is developed on the basis of the area under the receiver operating characteristic (ROC) curve. It estimates the probability that the predicted results are in accordance with the actual observed results:

(3) $$\begin{array}{c} C_{\text{index}}=\frac{\sum_{k=1}^{n}S_{k}\left(X_{i}\right)}{N}. \end{array}$$

Among them, *N* is the number of pairs that can be compared, and *S*_*k*_(*X*_*i*_) is based on whether the prediction result is consistent with the actual survival time.

(4) $$\begin{array}{c} S_{k}\left(X_{i}\right)=\left\{\begin{array}{c} 0,\;\text{not consistent,}\\ 1,\;\text{consistent,}\\ 0.5,\;\text{other}. \end{array}\right. \end{array}$$

The larger the *C*-index, the better the performance of the model. Note that 0 ≤ *C*-index ≤ 1 and that *C*-index = 0.5 corresponds to a procedure doing no better than random guessing, whereas *C*-index = 1 indicates perfect accuracy.

## 3. Results

### 3.1. Selected Predictors

The univariable Cox analysis revealed which of the correlation predictors were significant. There were 139 predictors (*P* value < 0.05) which were screened from 8,459 variables. In addition, the number of predictors decreased to 54 in the multivariable Cox analysis.

**Figure 1:**
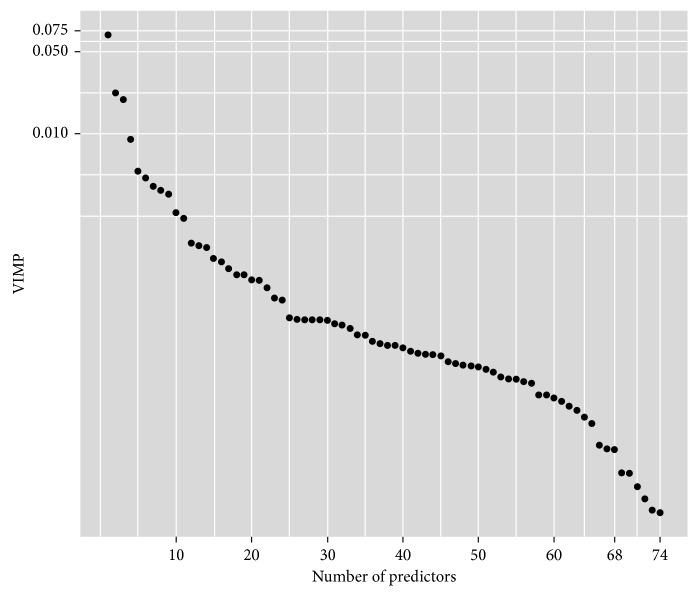
VIMP for predictors with VIMP > 0.

The VIMP of each predictor was calculated in Cox-VIMP, and for 74 predictors, the VIMP was >0. Those predictors were sequenced from high to low by VIMP, as in Figure 1. Predictors with VIMP < 0 were treated as not important and not drawn in the diagram.

**Figure 2:**
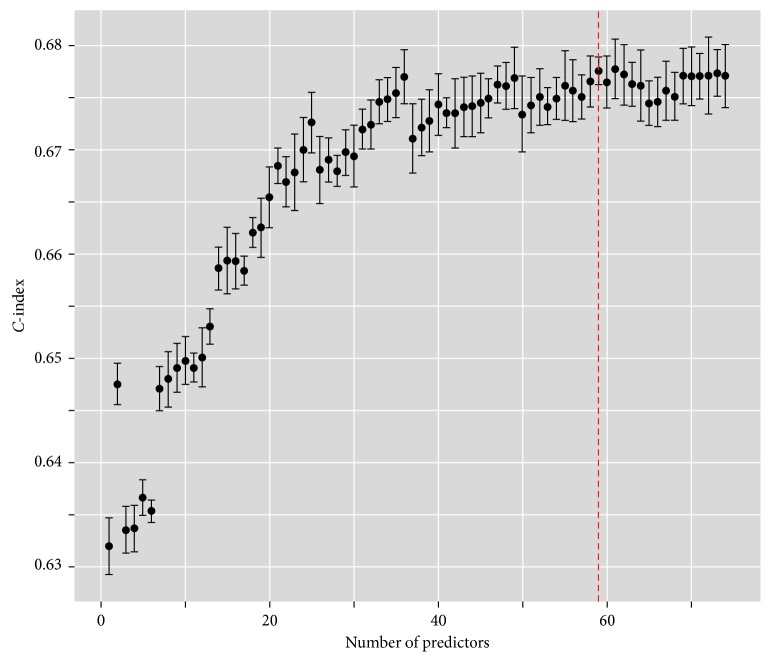
Average *C*-indexes of nested analysis after 5-fold cross validation.

The 74 predictors sequenced by VIMP can be used for nested analysis. Thus, we conducted nested analysis using the first 74 predictors (i.e., we obtained 74 prediction models). The 5-fold cross-validation method was used to test the stability of those 74 models. We used the average *C*-index as the final *C*-index after cross validation (represented in Figure 2 by dots). The upper and lower are mean plus standard deviation and mean minus standard deviation, respectively.

The maximum *C*-index minus standard deviation will be available (pessimistic principle), which corresponds to the red dotted line. Thus, the prediction model with the top 59 predictors was treated as the best model.

In the intersection scheme, the 29 predictors that were selected from the previous three schemes were used to build the RSF model.

The predictors were classified into three categories: (1) non-disease predictors, containing demographic and clinical predictors, (2) diagnosis predictors, and (3) surgery predictors.

**Figure 3:**
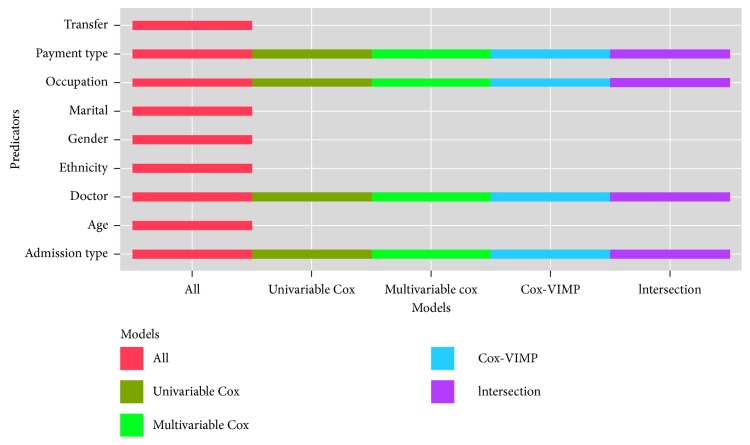
Non-disease predictors in the four schemes.

The non-disease predictors in each scheme are shown in Figure 3.

It is obvious that there are similar non-disease predictors in the four schemes, involving payment type, occupation, doctor, and admission type.

**Figure 4:**
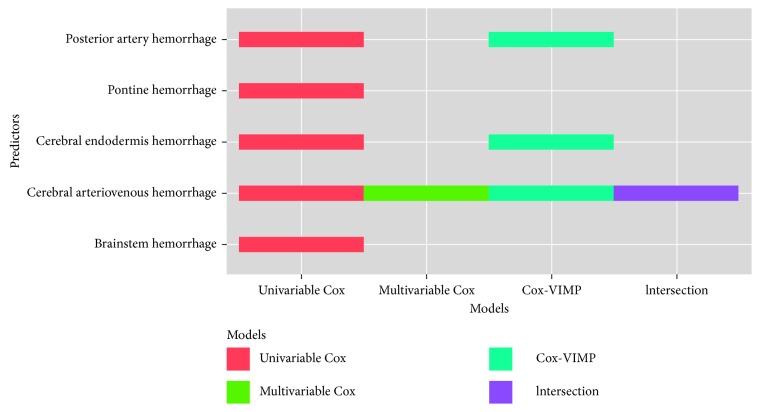
Main diagnosis predictors in each scheme.

**Figure 5:**
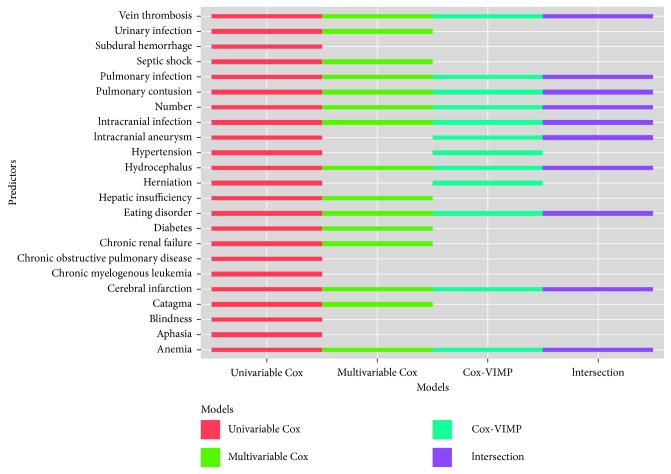
Preexisting diseases diagnosis predictors for each scheme.

Diagnosis predictors involved two parts: the main diagnosis showing the hemorrhage location and the other indicating preexisting diseases. For many diseases under the same type in medicine, we divided predictors into different types, following suggestions from doctors. The main diagnosis predictors are shown in Figure 4, and the preexisting diseases predictors are shown in Figure 5.

The *Y*-axis represents the significant hemorrhage location predictors. There are 5 significant hemorrhage location predictors in univariable Cox, 1 in multivariable Cox, 3 in Cox-VIMP, and 1 in intersection.

The results of preexisting diseases diagnosis predictors are given in Figure 5.

The *Y*-axis represents the significant preexisting disease predictors. “Number” represents the number of diseases suffered by patients. There were 22 preexisting disease predictors found by univariable Cox analysis, 15 by multivariable Cox analysis, 12 by Cox-VIMP analysis, and 10 by an intersection analysis. In addition, the number of diseases that each patient was suffering from was treated as a significant predictor in our study.

**Figure 6:**
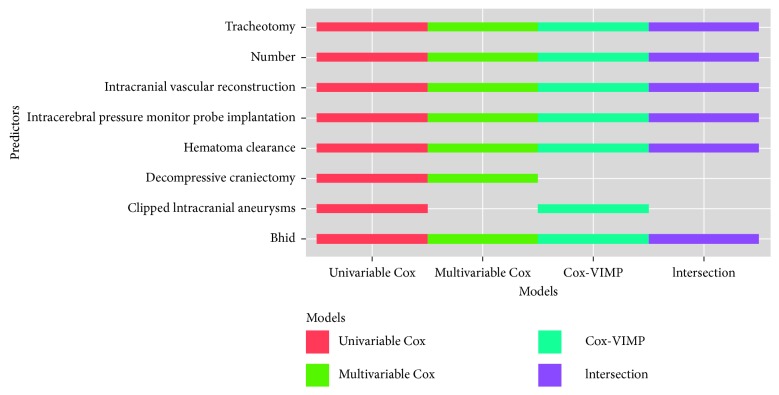
Surgery predictors of each scheme.

Surgery predictors could be categorized into two types: the contents of the surgery and the number of surgeries. The consequents are shown in Figure 6. We were able to directly sum up predictors and the number of surgeries.

Note that the surgery information is not the surgeries patients had undergone during the hospitalization, but the surgery details of the first operation after admission. The total number of surgery contents was 7,933. After selection of predictors, there were seven types of surgery contents in univariable Cox, six types in multivariable Cox, six types in Cox-VIMP, and five types in intersection. All schemes considered the number of surgeries as highly significant.

### 3.2. Prediction Results

**Figure 7:**
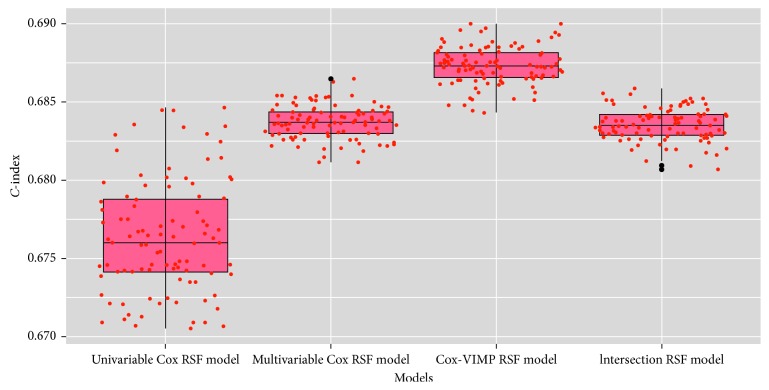
*C*-index box plot of each model.

Next, we used these sets of predictors to build four RSF models and ran each model 100 times with 80% randomly selected samples as the training set each time. The 100 *C*-index results of each model are represented as a box diagram in Figure 7.

Even if the sample was randomly selected each time, the results did not significantly change, which means that these models are stable and repeatable. The Cox-VIMP RSF model was chosen on the basis of the principle that the model with the maximal *C*-index is the optimal model. The mean *C*-index of the 100 experiments is provided in Table 2. We used a paired sample *t*-test to test whether there were significant differences between the models (Table 2). The original hypothesis was that there would be no significant differences between the models. If there were no significant differences between the models, the model classification would be invalid.

For all four models, the *C*-index was more than 0.6, which indicates that predictor selection can improve the prediction accuracy of models. The mean *C*-indexes of the four models were 0.6763 (univariable Cox RSF model), 0.6834 (multivariable Cox RSF model), 0.6873 (Cox-VIMP RSF model), and 0.6835 (intersection RSF model). When the performance of the model was judged by the average of the *C*-indexes, model 3, with the highest *C*-index, was determined to be the optimal model.

The results of the *t*-tests suggest that there was no significant difference between the multivariable Cox RSF model and the intersection RSF model, and that there were significant differences between other models. The predictors in the intersection RSF model were generated from the intersection of other model predictors, which explains why there were no differences between the multivariable Cox RSF model and the intersection RSF model with similar predictors. However, this result did not affect the conclusion that the Cox-VIMP RSF model was the optimal model, because there were significant differences between the Cox-VIMP RSF model and the other models.

## 4. Discussion

Health services research has emphasized the prediction of LOS probability distribution. In this paper, we selected the predictors with significant correlation for LOS probability distribution and constructed four RSF models based on four predictor-selection schemes. Consequently, predictors were eliminated and prediction accuracy was improved. Comparing the results of the four RSF models' *C*-index and a sample *t*-test for *C*-index, the Cox-VIMP RSF model is the best performing model and is significantly different from the other three models. Thus, the Cox-VIMP is the most useful model to select significant predictors.

There were similar demographic and clinical predictors in these four models. These were occupation, payment type, doctor, and admission type. Occupation was the only significant predictor in the category of demographics. We found that the most common predictors in other diseases' LOS prediction [31, 32], such as age and sex, were not significant in our study. Moreover, other studies [6, 33] had verified that these predictors have low contribution to ICH's LOS prediction. Ohwaki et al. [6] showed the limited role of sex and age in LOS. Naidech et al. [33] also found the same results. In addition, few studies have tested the significance of ICH patients' occupation on LOS prediction. However, some studies have looked at occupation [34, 35], which proves that we cannot ignore its influence. The results of our study demonstrate that ICH patients' LOS is significantly affected by occupation.

After testing the significance of payment type, doctor, admission type, and transfer in the category of clinical predictors, we found that these were significantly associated with LOS. Payment type is related to the type of medical insurance, which has been found to be associated with the disease state in other research [36, 37]. In ICH patients' LOS studies [4, 38], the type of medical insurance has no association with LOS, which is contrary to our results. The reason may be that we did not distinguish the type of medical insurance in our study and the types of payment in this paper were whether medical insurance or special medical insurance were used. In most cases, cash-payment patients are more eager to shorten their LOS and reduce costs than insurance patients. Patients with special medical insurance are more likely to receive quick service, which causes differences in LOS between other patients. It is controversial to regard the doctor as a predictor [39]. Little research has been conducted on this; however, Russell et al. [4] also confirmed that the different doctors predictor had an impact on ICH LOS. In fact, the type of physician determines the type of focus placed on the patient. Therefore, the difference in doctors can reflect the difference in the patients' disease condition.

The difference between the four model predictors is admission diagnosis and surgery information. In fact, some studies [6, 33] discussed whether hemorrhage location has a significant effect on LOS prediction. Naidech et al. [33] have shown that different hemorrhage locations affect LOS. Ohwaki et al. [6] found that hematoma location in the supratentorial or subtentorial regions has little influence on the ICH patients' LOS. However, in our study, we found that three hematoma locations are significant in ICH patients' LOS prediction. The reason for the difference from other studies is that we considered more predictors.

Meanwhile, preexisting diseases, such as hypertension, have significant influence on LOS prediction [4]. Marco et al. [40] discussed the comorbidities: arterial hypertension, diabetes mellitus, hypercholesterolemia, and atrial fibrillation, which had also been tested in our research. Specogna et al. [41] used 14 preexisting diseases in their model and considered 5 as significant: cardiac disorders, chronic pulmonary disease, mood disorders, peripheral vascular disease, and peptic ulcer disease. These studies provided proof that information on preexisting diseases is important. Our study used more information on preexisting diseases and had more significant predictors. The number of diseases related to LOS probability distribution is a predictor not considered in other studies. Consequently, doctors can use these predictors to take precautionary measures for patients. When patients with the same hemorrhage location are treated, doctors can predict LOS according to the information on preexisting diseases.

With regard to surgical information, we found some differences among the four models. The effect of surgery on LOS prediction has been confirmed [6, 13], which proves that we cannot ignore this information. Blanco et al. [42] studied the relationship between LOS and open abdominal aortic revascularization surgery. Ohwaki et al. [6] examined surgical intervention as an indicator, but not the surgical details. Few studies have examined the impact of surgical contents on ICH patients' LOS prediction. Our study explored the effect of the number of surgeries and the contents of surgery on LOS probability distribution.

Unlike other ICH patient LOS prediction studies, we considered the importance of LOS probability distribution, making full use of patient information. However, there are some limitations to our study. First, the accuracy of the prediction in our study can be improved. Second, we only used data from one hospital. It would be meaningful to compare the results of different hospitals. Third, we only predicted the LOS of one disease. Though a particular unit may only treat one disease, there are many diseases being treated within one department. In other words, our results may be more applicable to unit BM than to the BM of the department. In the next study, we will consider these limitations and expand the research.

## 5. Conclusion

This study sought to predict the probability distribution of ICH patients' LOS. We emphasized and made full use of diagnostic and surgical information, building Cox combination models to select predictors. As results, the significant correlation predictors were selected and LOS probability distribution was predicted with the Cox-VIMP RSF model. Through the results of our research, doctors gain a better understanding of which diseases and surgical contents affect LOS and better comprehend the LOS probability distribution of each patient. The patient's admission and discharge plans can then be effectively implemented.

## Figures and Tables

**Table 1:** Patient data.

*Time*	
Length of stay: mean, median	Mean: 12.61 days, median: 10 days

*Demographics*	
Gender	Male (53.5%), female (46.5%)
Age: mean, median	Mean: 53.79 years, median: 54 years (20 years)
Marital status	Single (6.8%), married (85.5%), divorced (2%), deceased (5%), other (0.8%)
Occupation	Unemployed (3.6%), employed (civil servant, student, farmer, worker, and so on) (96.4%)
Ethnicity	Han (94.3%), Tibetan (4%), other (1.7%)

*Clinical predictors*	
Payment type^1^	General medical insurance (26.8%), non-medical insurance (61.9%), special medical insurance (11.3%)
Doctor^2^	132 doctors
Admission type	Emergency (89.6%), outpatient (7.5%), other (0.8%)
Transfer^3^	Yes (24.7%), no (71%), unrecorded (4.3%)

*Admission diagnosis*	
ICD-10 diagnosis	The total number of hemorrhage locations and preexisting disease is 550
Diagnoses number^4^: mean, median	3, 2

*Surgery information*	
Surgery contents	The total number of surgery contents is 7,933
Surgery number^5^: mean, median	3.6, 3

ICD 10 = 10th revision of the International Statistical Classification of Diseases and Related Health Problems. ^1^General medical insurance is a common type of medical insurance; special medical insurance means that there are some green channels to pay faster and receive services more quickly. ^2^Number of attending doctors in this department. ^3^Whether or not the patient was transferred from another medical institution. ^4^Number of main diagnoses and other diagnoses for each patient. ^5^Number of surgeries undergone by each patient. Note that the surgery information is not on the surgeries patients had undergone during hospitalization but on the surgery details of the first operation after admission.

**Table 2:** The *C*-index of each model.

Model	Mean	SD	Paired sample *t*-test (*P* value)			
			Model 1	Model 2	Model 3	Model 4
Model 1: univariable Cox RSF model	0.6763	0.0036	—	<0.001^*∗*^	<0.001^*∗*^	<0.001^*∗*^
Model 2: multivariable Cox RSF model	0.6834	0.0012	—	—	<0.001^*∗*^	0.336
Model 3: Cox-VIMP RSF model	0.6873	0.0012	—	—	—	<0.001^*∗*^
Model 4: intersection RSF model	0.6835	0.0010	—	—	—	—

RSF = random survival forests; SD = standard deviation; VIMP = value of importance. The original hypothesis was that there would be no significant differences among the models in the *C*-index.^*∗*^*P* value < 0.01.

## Data Availability

The data used to support the findings of this study are restricted by the West China Hospital in order to protect patient privacy. Data are available from West China Hospital for researchers who meet the criteria for access to confidential data.
